# Supplementary figures and images for: Microglial activity during postnatal development is required for infantile amnesia in mice
Source: PLoS Biol. 2026 Jan 20;24(1):e3003538. doi: 10.1371/journal.pbio.3003538 (PMC12818610; doi:10.1371/journal.pbio.3003538)

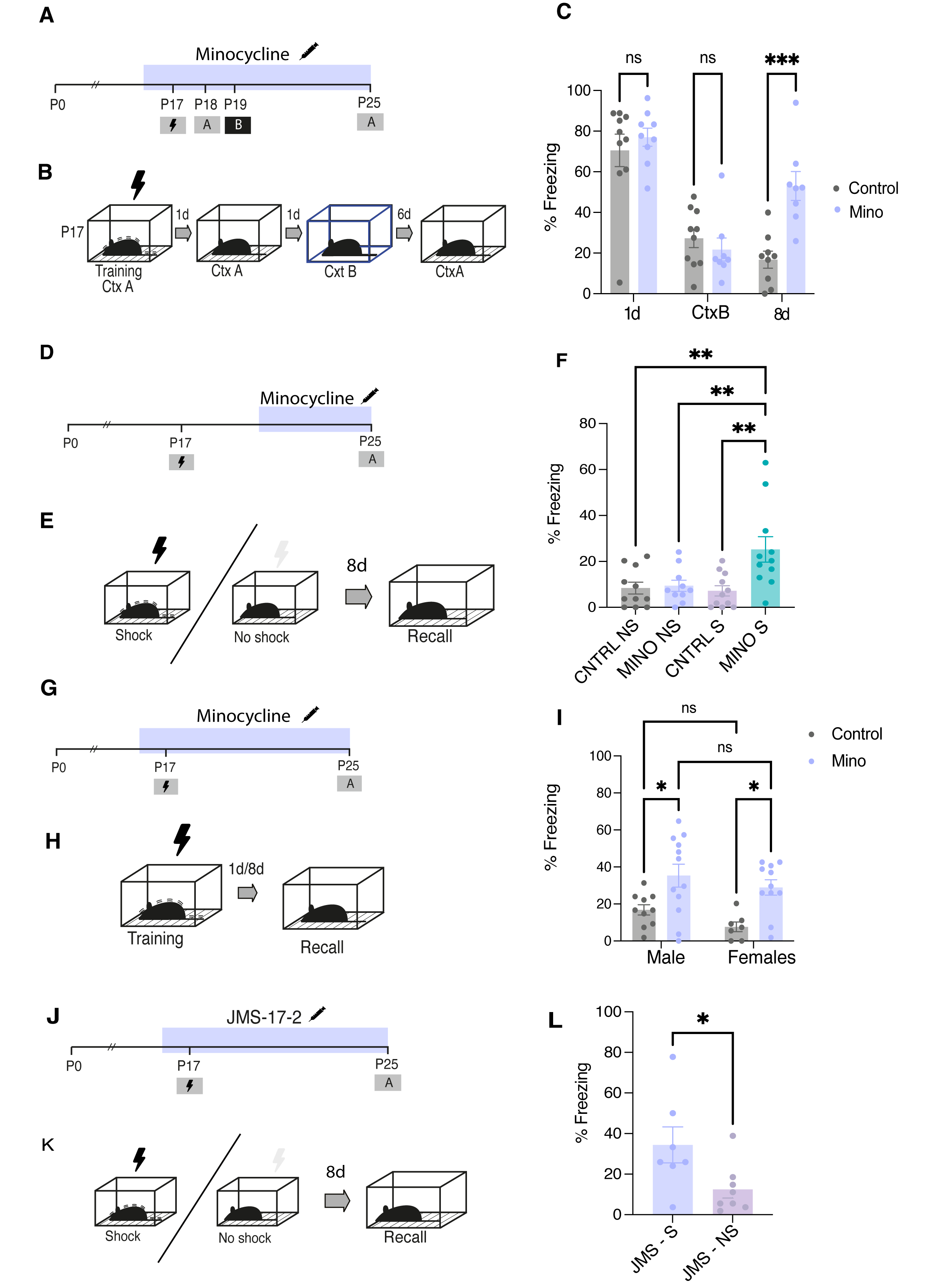

Supplement: S1 Fig — (A) Schematic diagram of experimental schedule. Mice were injected with 50 mg/kg minocycline (mino) or control from P16-P25. (B) Schematic of behavioral schedule. Mice were trained at P17 and underwent recall test 1 day (Test 1) and 8 days (Test 3) post-training. Mice underwent a generalization test in Context B at P19. (C) Quantification of freezing behavior. (D) Schematic diagram of experimental schedule. Mice were injected with 50 mg/kg mino or control from P20-P25. (B) Schematic of behavioral schedule. Mice were trained at P17 (S) or received context exposure with no shock (NS) and underwent recall 8 days post-training. (F) Freezing behavior of mice that received either S or NS and mino treatment or control. (I) Comparison of freezing behavior between male and female mice treated with minocycline or control 1 and 8 days post-training. (J) Schematic diagram experimental schedule. Mice were administered JMS-17-2 (10 mg/kg) through i.p injection from P16-P25. (K) schematic of behavioral schedule. Mice were trained on P17 and tested for recall 8 days later. (L) Quantification of freezing behavior between shock (S) and no-shock (NS) JMS-treated mice. Black lightning symbol represents foot-shocks and empty lightning symbol indicates no-shock. Data is presented as mean ± SEM. (n = 8–11). (C, I) Statistical comparison performed using RM Two-way ANOVA with Bonferroni; (F) One-way ANOVA with Bonferroni; (L) Student’s t test; n.s P > 0.05, *P < 0.05, **P < 0.005. Details of all statistical comparisons may be found in S1 Data. The data underlying this Figure can be found in S2 Data. (TIF) [file pbio.3003538.s001.tif]

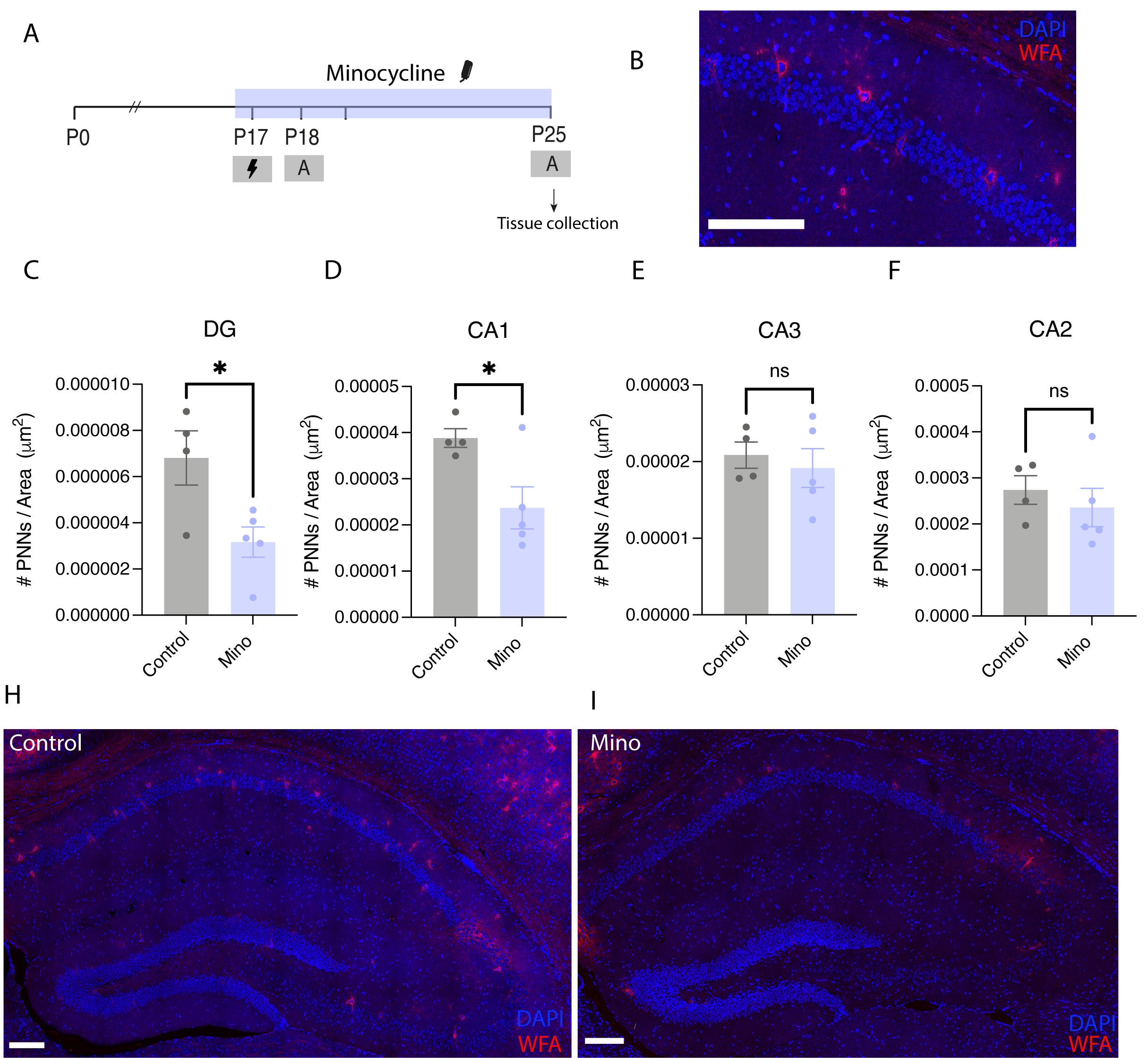

Supplement: S2 Fig — (A) Schematic diagrams of experimental schedule. Mice were administered minocycline (50 mg/kg) through drinking water from P16-P25. Following recall on P25 mice were sacrificed and transcardially perfused for brain tissue collection. (B) Representative images of hippocampal subfields analyzed for WFA staining. Scale bar = 150 µm. (C–F) Quantification of PNNs per area in DG, CA1, CA2, and CA3. (G–H) Representative images of WFA staining in controls and minocycline-treated mice. Scale bar = 75 µm. (n = 4/5). Each point represents an individual mouse. Statistical comparison performed using Student t test; n.s P > 0.05, *P < 0.05. Details of all statistical comparisons may be found in S1 Data. The data underlying this Figure can be found in S2 Data. (TIF) [file pbio.3003538.s002.tif]

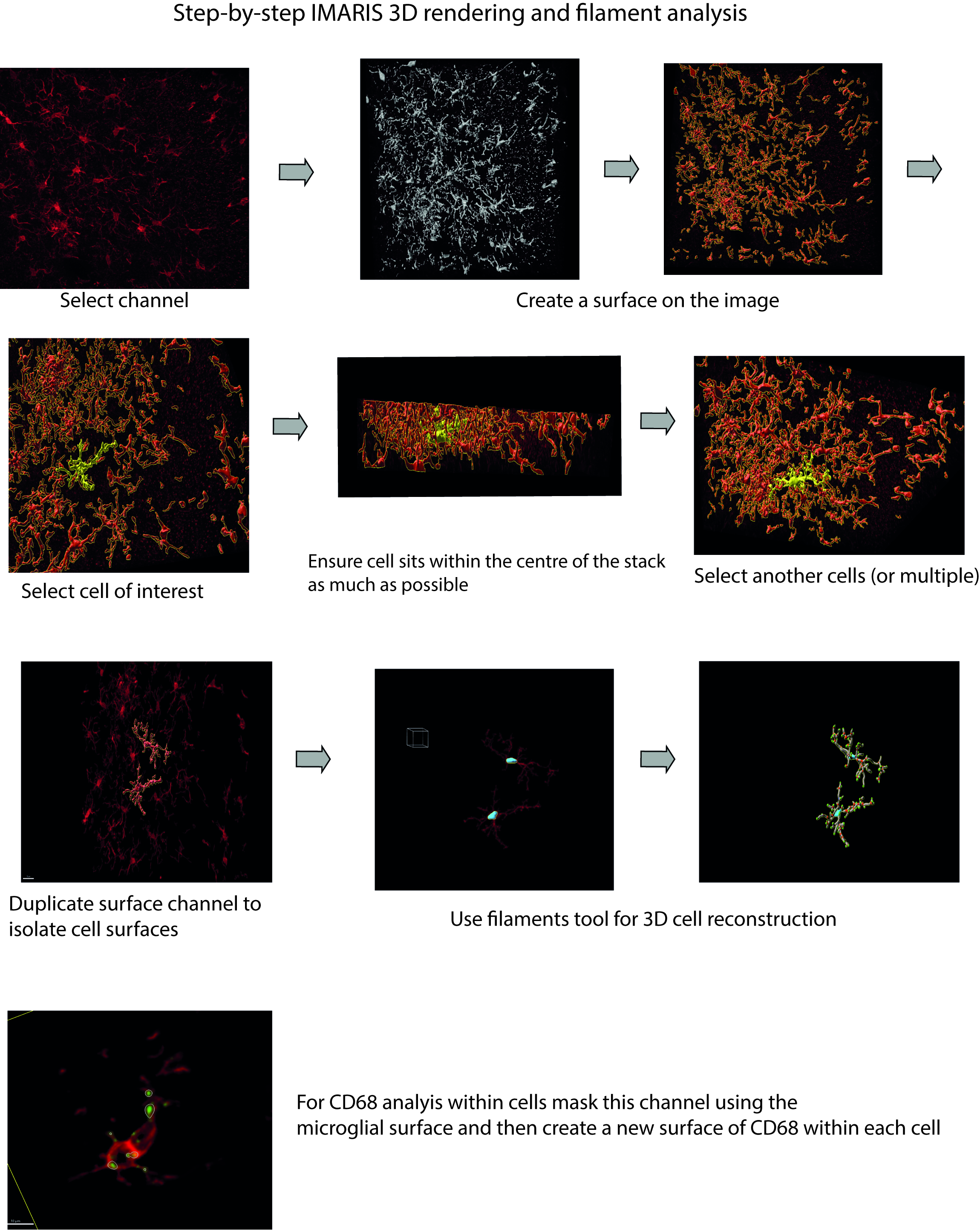

Supplement: S3 Fig — Step-by-step example of IMARIS 3D rendering and filament analysis. The IMARIS “surface” function was used to create surface of each channel of interest followed by selection and isolation of cells of interest. The IMARIS ‘filaments’ function was used to trace and construct models of individual microglial cells and subsequently quantify filament length, branch points, and terminal points. For quantification of CD68 expression within microglial cells, the microglia surface was used to create a mask of CD68 channel and create a new surface of this masked channel. The volume of CD68 surface within each volume of microglia surface can be used to calculate relative CD68 expression. (TIF) [file pbio.3003538.s003.tif]

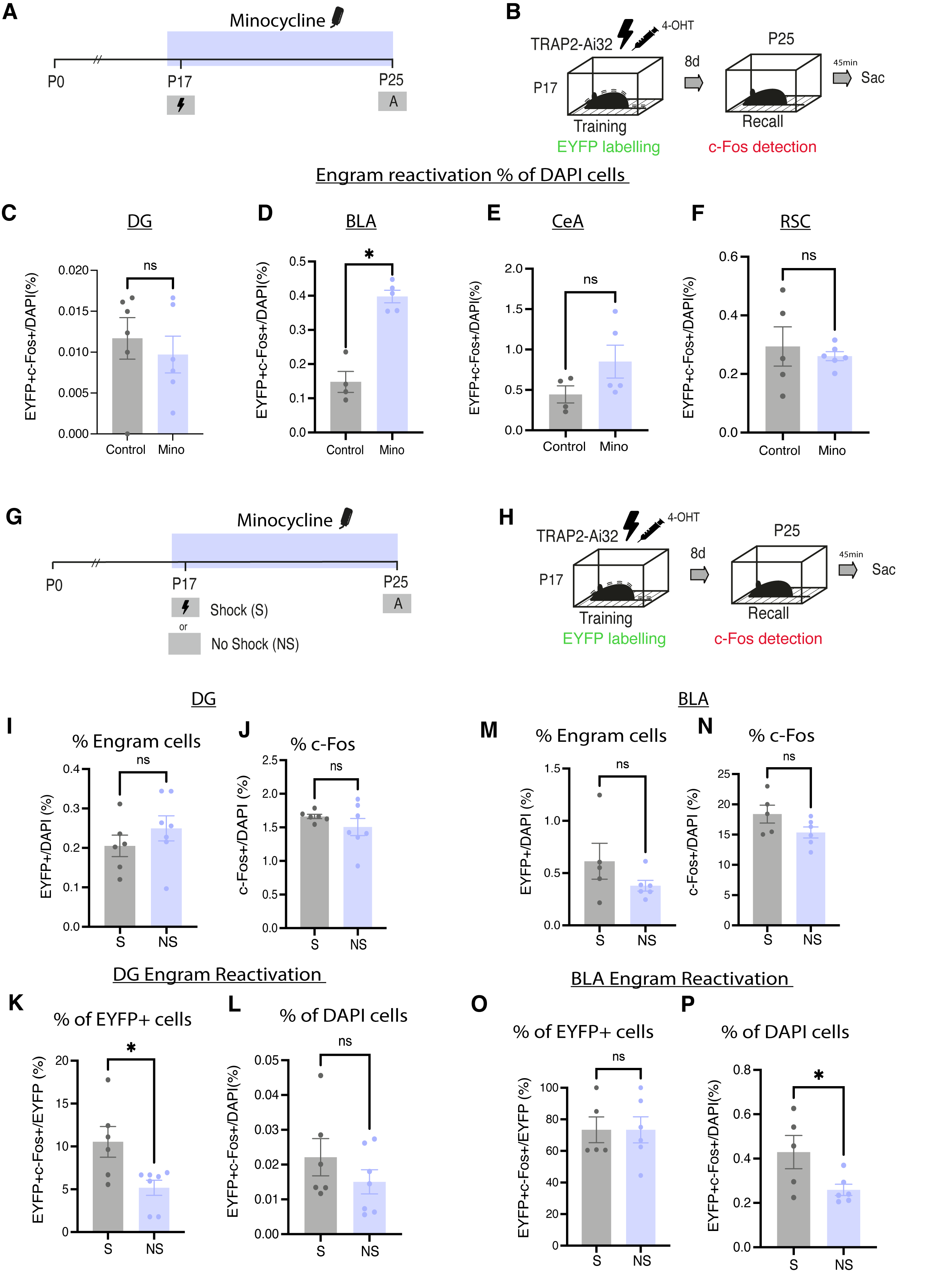

Supplement: S4 Fig — (A) Schematic diagram of experimental schedule. Mice were administered 50 mg/kg minocycline from P16-P25 through drinking water. (B) Schematic of behavioral schedule. Syringe symbol represents 4-OHT injection. Black lightning symbol represents foot-shocks. (C–F) Quantification of % engram reactivation (EYFP+c-Fos+/DAPI) in control versus minocycline-treated mice. (G, H) Mice were trained at P17 and underwent either CFC (S) or Contextual exposure (NS) and mice underwent a recall test 8 days later. (I–L) Quantification of % Engram cells, % c-Fos, and engram overlap/reactivation in DG. (M–P) Quantification of % Engram cells, % c-Fos, and engram overlap/reactivation in AMG. N = 5–7 mice/group, n = 4 slices per mouse. Data is presented as mean ± SEM. Each point represents individual mice. Statistical comparison performed using Student’s unpaired t test; *P < 0.05. Details of all statistical comparisons may be found in S1 Data. The data underlying this Figure can be found in S2 Data. (TIF) [file pbio.3003538.s004.tif]

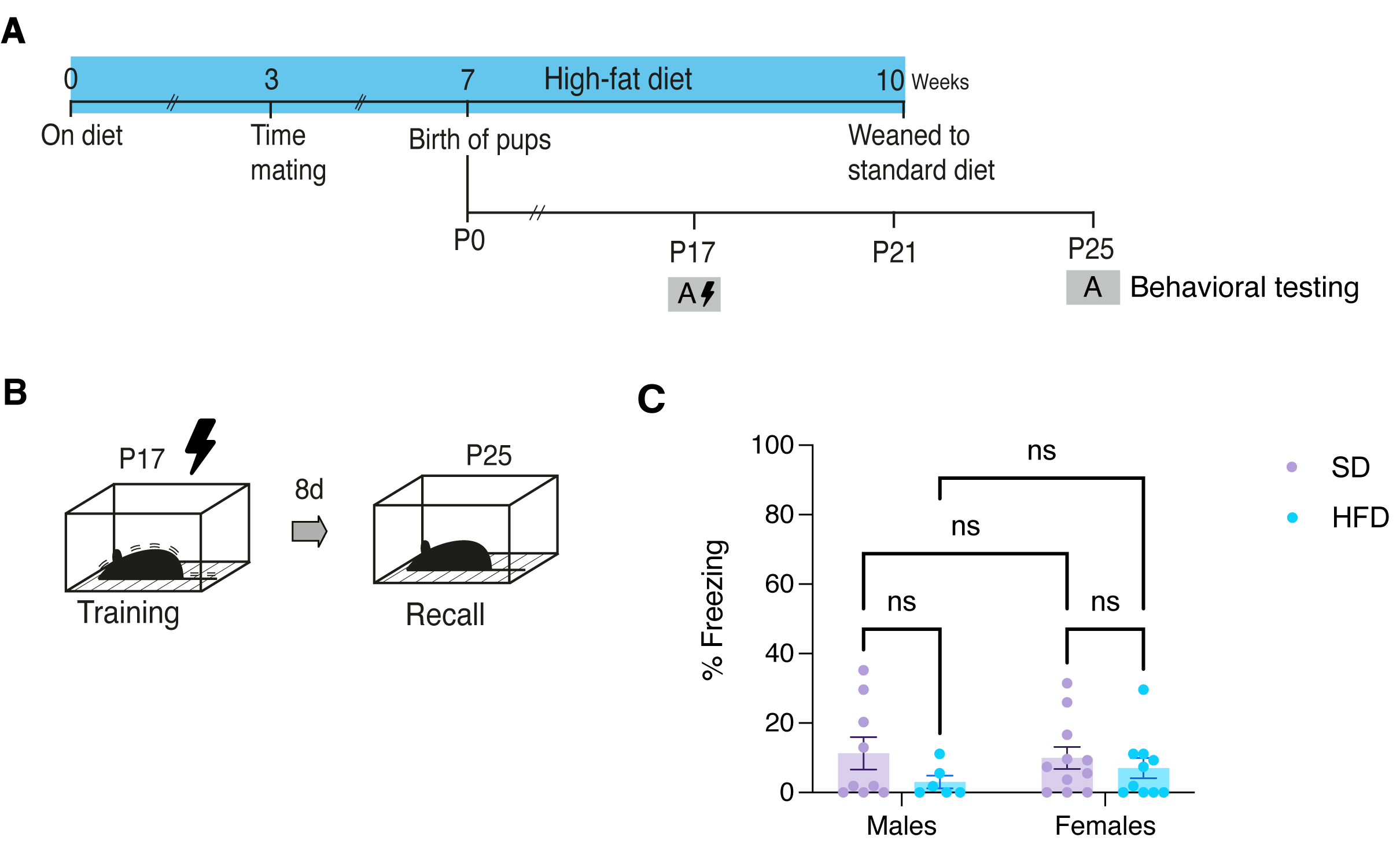

Supplement: S5 Fig — (A) Schematic diagram of experimental schedule. Female mice are placed on a high-fat diet (HFD) 3 weeks prior to time-mating and remain on the diet through gestation and lactation until offspring are weaned at P21 while control mice remain on standard diet (SD). (B) Schematic of training and testing schedule. Male and female offspring underwent CFC at P17 and were tested for memory recall 8 days later. (C) Freezing behavior of mice during recall test. Data is presented as mean ± SEM. (n = 6–12) mice/group. Statistical comparison performed using Two-way ANOVA with Bonferroni; n.s P > 0.05. Details of all statistical comparisons may be found in S1 Data. The data underlying this Figure can be found in S2 Data. (TIF) [file pbio.3003538.s005.tif]

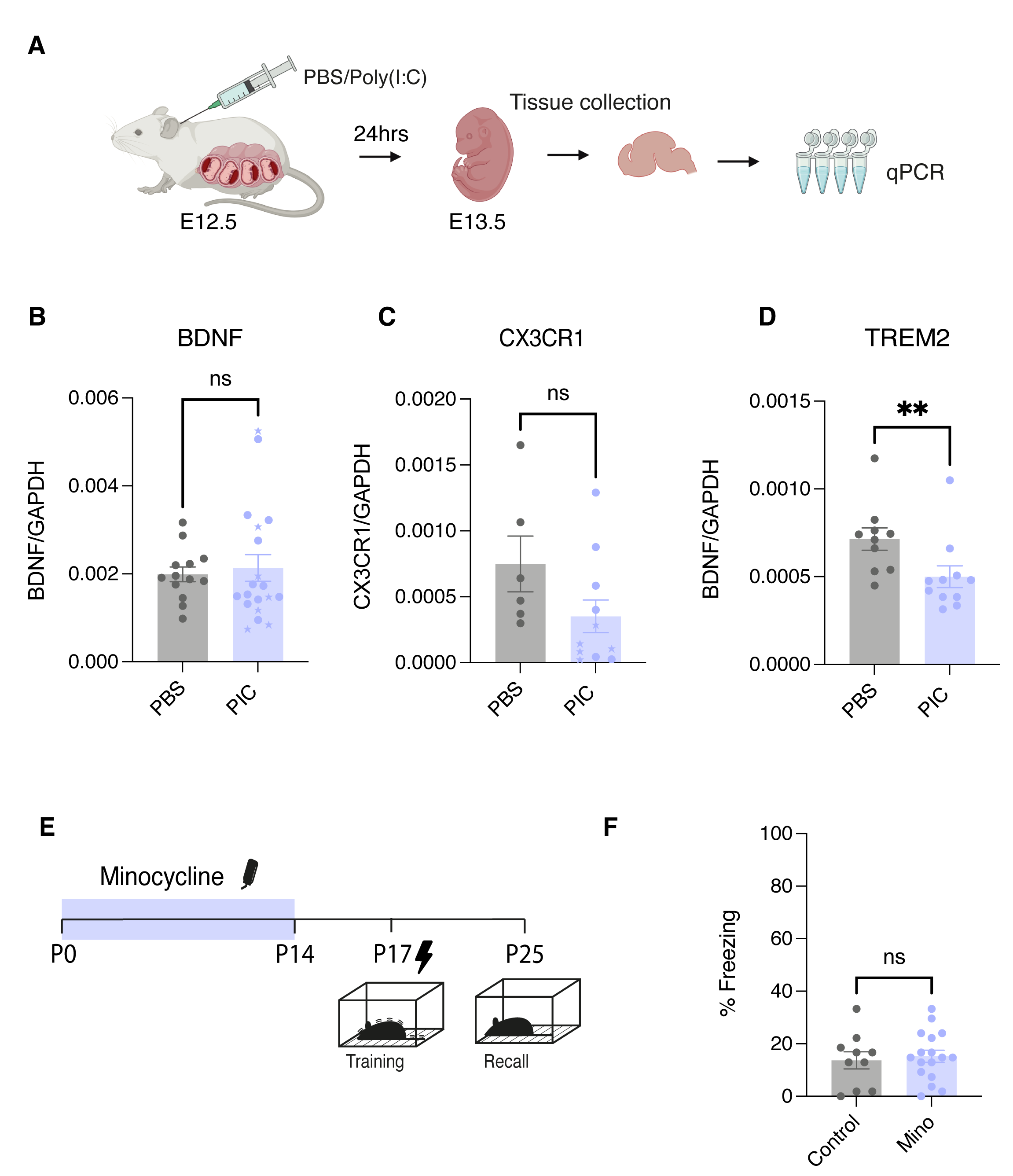

Supplement: S6 Fig — (A) Experimental scheme. Pregnant female mice were injected with either PBS or Poly(I:C) at gestational day 12.5. Twenty-four hours following injection mice were deeply anaesthetized and embryos were extracted. Embryo brains were collected and snap frozen until use. RNA was extracted from brains, cDNA was generated, and qPCR was performed to quantify mRNA expression. (B–D) Relative expression of microglia-related mRNA between embryos from PBS or Poly(I:C) injected dams. Created in BioRender. Stewart, E. (2025) https://BioRender.com/pmb4ujv. (B) Relative mRNA expression of BDNF (n = 13/17). (C) Relative mRNA expression of CX3CR1 (n = 6/11). (D) Relative mRNA expression of TREM2 (n = 10/11). (E) Schematic diagram of experimental schedule. (F) Quantification of freezing in WT control mice treated with minocycline or vehicle from P0-P14. Data is presented as mean ± SEM. Statistical comparison performed using (B–D) Mann–Whitney test and (F) nest t test. n.s P > 0.05. Details of all statistical comparisons may be found in S1 Data. The data underlying this Figure can be found in S2 Data. (TIF) [file pbio.3003538.s006.tif]
