# Supplementary material for: Microglial activity during postnatal development is required for infantile amnesia in mice
Source: PLoS Biol. 2026 Jan 20;24(1):e3003538. doi: 10.1371/journal.pbio.3003538 (PMC12818610; doi:10.1371/journal.pbio.3003538)
Supplement: Attachment — Submitted filename: Response to Reviewers.docx [file pbio.3003538.s011.docx]

**Response to Reviewers**

Reviewer #1: Summary this study reveals a novel role for microglia in regulating memory accessibility during early life, offering new insight into the mechanisms of infantile amnesia. By profiling microglial changes across postnatal development, the authors show that inhibiting microglial activity prevents infantile forgetting of a contextual fear memory. Using activity-dependent tagging, they demonstrate that microglial inhibition alters engram size, reactivation, and interactions in the amygdala. Notably, microglial dysfunction was linked to persistent early-life memory in maternal immune activation (MIA) offspring. These findings position microglia as active modulators of infant memory and highlight their relevance in neurodevelopmental disorders. There are a few areas of improvement that should be addressed prior to publication.


Major Concerns:

1.      For Figure 1 B and C, it would be helpful to show the initial training responses to each shock. How were the shock intensities chosen? Were they the same for the two ages? Ideally, the immediate shock response and freezing would be the same at training across age. Please also make it clear if the same animals were tested repeated across days or different animals were tested for each day. If the same animals were retested, how was extinction accounted for? If different animals were tested, please remove the dotted line between timepoints as it implies a repeated test of the same animals. It is also a bit confusing to use postnatal day for B and time post testing for C.

We have now presented the initial freezing responses of the mice of all age groups (Fig 1B). All infant mice were trained at P17, and adults at P56. We demonstrate that infant mice trained show a higher initial response to the first shock compared to adults but show similar levels to adults after the second and third shock and show no significant differences at baseline or on average across the 3 shock minutes.

The shock intensity chosen was 0.75 mA (a standard current intensity chosen based on our previous work). This intensity produces robust fear memory formation as illustrated in the high freezing observed in all groups during a recall test 1d post training which is indicative of fear memory. Separate groups of mice were used for each time point to prevent the effect of extinction. We have removed the dotted line connecting groups and present groups as separate bar charts. We now also use time post testing (1, 5, or 8 d) for both infants and adult groups.

2.      Only 3-5 microglia were analyzed per brain region per mouse. How were these microglia selected? Was the experimenter blinded to treatment conditions?  How was it determined that only 3-5 cells was representative of the microglia within an entire region given the large and well documented heterogeneity in microglia? Same comment for the microglial co-localization with CD68 analysis.

We thank the Reviewers for pointing this out. This was a mistake in the figure legend regarding the number of cells analysed for each mouse. For each animal, 2 ROI images were taken and 3-5 microglial cells were analyzed per ROI. Cells were then pooled for each animal. We have updated the figure legends accordingly.

The experimenter was always blinded to treatment conditions during analysis.

There is well documented microglial heterogeneity within the hippocampus and even within the DG itself, particularly between layers. We focused specifically on microglia within the molecular layer of DG. Furthermore, to reduce bias as much as possible we followed certain criteria when selecting the cells. We selected cells that sit in the middle of the stack image with the least amount of processes cut off. Once these conditions are considered there is typically 3-5 microglia cells (maximum) in a given image that fit these criteria and that reduces objective bias substantially. We have also updated the methods section to include a detailed, step-by-step illustration and example images of cell selection and analyses.

3.      For all figures, please specifically denote males and females with different dot colors or shapes where both sexes were used. Clearly denote when just male mice were used.  Where both sexes are used, sex should be included in the statistical analysis as a main effect and interaction. If sex was not included in the majority of the mechanistic studies, consider adding females for a subset of measures or discussing this as a limitation in the discussion and conclusions.

Where males and females are included we have changed the graph to denote males and females with different symbols. In the minocycline treatment there was a main effect of treatment (minocycline), no effect of sex and no interaction. We include a stats table and note statistical analysis in more detail which can be found in supplementary material and is noted in the results section.

We have also acknowledge in our discussion the limitation of some of our analysis for the sole use for males.

4.      There are several instances of the use of the phrase "microglial activation" (ie. line 203, 211 etc). It is unclear what exactly this means. The changes in morphology and contact with neurons suggest changes in microglia activity, which is not the same thing as activation, which in the literature implies immune activity or inflammation. Please clarify the exact meaning of activation and consider other word choices for clarity.

We agree and we have now replaced the word activation with “activity”.

5.      It is surprising that the Mino group showed reduced microglia-neuron contacts (Figure 3Z) while also showing more branching (3U-W). Were there any changes in the neuronal structure between groups that could help explain this?

We did not quantify dendritic branching in these groups however we did normalise for differences in engram surface between groups.

6.      It is unclear how MIN can both facilitate amnesia (Figure 4B) in MIA and prevent amnesia in WT mice (Figure 2C). Similarly, in MIA MINO increases CD68 in microglia (Figure 4G) and in WT MINO decreases it (Figure 2E). One obvious difference is the timing of MINO treatment between the two experiments. Please add a WT control condition to Figure 4 to clarify the impact of MINO on VEH treated animals under the same conditions as MIA.

We thank the Reviewers for this comment and we have now added this control condition. WT control mice received either MINO or VEH during the same time frame as MIA mice (P0-14) (Figure S5). This had no effect on infantile amnesia in control mice. This helps clarify that microglia inhibition has discrete affects on infantile amnesia and the development of

MIA, depending on time of intervention.

Minor Concerns:

1.      Line 66: "…microglia are of the one candidate mechanisms…" should be "microglia are one of the candidate mechanisms"
2.      The text on lines 253-259 describes Figure 4H. There is no H in figure 4.
3.      Line352: crucial for establish accurate connectivity should be establishing
4.      Why was the RSC only included in the Figure 3 work and not in Figure 1?
5.      Figure S2 is not really connected to the rest of the paper. Figure S4 is not cited in the text and Figure S3 is not cited until the conclusions (out of order and missing from the results).

We thank the Reviewer for catching these errors and we have corrected them in the updated manuscript and we have changed the order of the supplemental figure to match how they appear in the text.

We originally did not include RSC as this region was not originally of interest to us. However. we came across the preprinted work Laura Denardo’s group (Jin et al., 2024) that identified RSC as a key player in the emergence of persistent memory. We then went back to our tissue to image and counted this area, however when we did not see any differences in engram activity or c-Fos in this region we did not continue with RSC investigation. While RSC may be important in persistent adult-like memory, our data suggest it is not involved in the retention of fear memories in our model.

Reviewer #2 (Alonso Martínez Canabal): The manuscript presented by Stewart et al. investigates the relationship between microglial activity and infantile amnesia—a characteristic of altricial mammals, including humans and many of the common rodent models used in biomedical research (though not all). This research is important and relevant because, despite the critical role of infantile amnesia in human cognitive and mental development, it has been historically neglected in neuroscience, particularly within the biomedical framework. While clinical psychology has paid somewhat more attention to the topic, the overall attention remains limited. This neglect has hindered our understanding of how infantile amnesia may relate to the development of neurodevelopmental disorders such as ASD and ADHD, as well as pediatric psychiatric conditions like early-onset depression or PTSD.

This study is also important for highlighting the role of microglia—a cell type often overlooked in cognitive neuroscience, which traditionally focuses on neurons and, to a lesser extent, astrocytes and oligodendrocytes. However, emerging research suggests that microglia play a more active role in brain development and circuit tuning than previously appreciated. The present study supports this notion by demonstrating a causal relationship between microglial activation and the persistence of memory formed during the preweaning period and tested shortly after weaning.

The introduction is well-written, and the Materials and Methods section includes sufficient detail for replication by researchers with appropriate technical expertise. However, there is an inconsistency in the Results section: on line 132, the text refers to a graph (freezing behavior), but the corresponding figure shows a microphotograph. This should be corrected.

We thank the Reviewer for catching this and have now corrected this in the results section.

The subsection "Inhibition of microglial activity alters infant engram dynamics" is particularly compelling and central to the study. It is intriguing that engram activation in the dentate gyrus (DG) does not show significant differences, which contrasts with findings from Guskjolen et al. (2018), where this was a major result. The authors attribute their main findings to the amygdala; however, they do not present the corresponding behavioral data. Although the experimental design includes contextual fear conditioning (CFC) training and recall, freezing behavior is not clearly reported—except in Panel X as part of a Pearson correlation with endpoint measurements. It is essential to present the behavioral data (as done in Figure 2C for Mino vs. control groups) to fully understand the relevance of the observed engram activity.

We thank the Reviewer for this comment. We have now plotted and displayed the freezing behaviours for T2-Ai32 mice treated with either minocycline or control (Figure 3X).

We do not see any discrepancy between our manuscript and the Guskjolen/Frankland paper. That paper showed higher engram reactivation in the DG (and other areas) only after optogenetic reactivation. We are showing natural recall here, and our data are consistent with maternal immune activation experiments in Power et al ’23 (cited in our manuscript), as we did not see any difference in engram reactivation in the DG compared to controls, only a difference in engram size. In addition, the Frankland paper used later time points to us, not P25.

In the Discussion, the authors cite Akers, Guskjolen, and Travaglia, who address both infant neurogenesis and electrophysiological maturation. It would also be appropriate to consider the work of López-Oropeza (2022). Despite these references, the discussion does not address the potential role of newborn neurons—known to be produced at a rate approximately seven times higher in infants than in adults—in the context of infantile amnesia. Given that previous neurobiological hypotheses have proposed a key role for these neurons, this omission should be rectified. Furthermore, while the paper briefly mentions pruning, it does not explore the potential interplay between microglia and newborn neurons, which could be central to the mechanisms underlying infantile amnesia. The authors should expand the discussion to consider how microglial activity may interact with increased neurogenesis during infancy, particularly in light of the literature already cited in the introduction.

We thank the Reviewer for bringing the relevant work of López-Orpeza to our attention. We have now discussed this paper in our discussion and have furthered our discussion to include the potential role of newborns neurons and how microglia relate to this process.

Reviewer #3: The authors investigate the role of microglia in episodic-like memory development by assessing morphometric changes and the effect of inhibiting their activity early in life on the expression of infantile amnesia and engram related activity. They observe that microglia may be causally involved in infantile amnesia, suggesting microglia-related maturational processes may be critical for healthy memory development, they argue their results has implications for our understanding of the cognitive deficits observed in maternal immune activation models.

The authors have done an impressive amount of work and the results would surely be of interest to the learning and memory neuroscience community as well as developmental researchers. That being said, I have a number of reservations regarding the morphological analyses carried out, the link between the different findings explained and how the results relate and advance the current state of the art.  I detail specific points below.

We thank the Reviewer for the positive appraisal.

        * The first section of the paper describes developmental morphological changes in microglia. The authors note significant changes in DG microglial processes between P20 and P25 (and between P20 and P22 in AMG) but also find changes in adult samples when microglia processes are compared between animals whose memory was tested at different retention intervals. This begs the question, do the developmental changes relate to development or the longer retention interval in the older animals?  It is difficult to answer this question with the current data. However, what the authors could analyse is how the different morphological markers change with the temporal extent of the retention interval (e.g. the adults show a reduction in branch points for more remote memories, is a similar temporal trend observed in development?).  This would highlight any developmental changes.  Further, they could comment on how the morphological markers differ in general between the pup and adult data.
Looking at Fig1, it seems all the morphological measures are lower in quantity in development. However, this point is not discussed.

In both cases, infant and adult, mice were trained and tested at different retention intervals (3d, 5d or 8d) but we thank the Reviewer for pointing this out as we believe it was confusing that we had labelled our infants groups as their age (P20, P22, P25) rather that recall day (3d,5d or 8d). We have updated Figure 1 to avoid this confusion.

Therefore in this case we are looking at how microglia change both as a function of development and memory retention - though we agree it is difficult to say whether the changes are purely developmental in nature or are related to the memory, our interest is in microglia activity post learning and changes that might indicate differences in activity in mice that remember versus mice that don’t. We know from the literature that microglia change as a function of development but nobody thus far has looked at how microglia change also at different retention intervals post memory formation. This was our goal although we agree that may not have been clear. Therefore we have also made some text changes to reflect this. Given that we have seen changes in infant that we do not see in adults with stable memory, we proceeded with further investigation to examine where these observed changes might play a role in infantile amnesia.

The lower quantify of morphological features between infant and adult mice is expected because microglia are maturing during this time and so lower quantify of morphological features is well known (Dalmau *et al.*, 1998; Orłowski, Sołtys and Janeczko, 2003). We have now commented on this difference in the results section.

        * The link between this first section is only superficially linked to the subsequent section(s).  Namely, the subsequent sections effectively show that inhibiting microglia early in life leads to accelerated memory development (memories formed at P17 are retained at P25), suggesting perhaps microglia hyperactivity  early in life may play a causal role in infantile amnesia, and thereby healthy memory development. Yet, the first section does not address overall activity of microglia in development, rather it focuses on specific morphological features. This makes it difficult for the reader to link the different sections and to understand what the mechanistic link between microglia maturation and memory maturation is. Could the authors simply quantify microglia activity in development? This would help create more coherence between the different sections and help clarify the take-home message.  Further, it would be useful if the authors used the discussion to synthesise better their different experimental findings.

We thank the Reviewer for this comment and as suggested we have now quantified microglial activity across development through analysis of CD68 puncta within microglial cells to give us a better picture of activity across development and we agree this add substantially to our findings and provides a better link between the different sections (Fig. 1). We have also added to our discussion to better synthesise our findings.

        * The second results section of the paper shows inhibiting microglial activity (via minocycline administration) prevents infantile amnesia. This is an interesting and novel result.  However, the authors found paradoxically that PNNs were sparser in the minocycline treated mice.  These results seem to contradict a paper published a couple of years ago (Ramsaran et al. (2023)) showing PNNs increase in expression in development and this increase is associated with memory maturation.  Here, the authors essentially accelerate memory development but when they do this they see a reduction in PNNs.  Could the authors please try to address this inconsistency. Do the authors think the mechanisms that underlie the precocious emergence of memory in the minocycline mice is mediated by different neurobiological processes than natural memory development?

We appreciate that this appears contradictory, however, we do not assume the retention of the infant memory that we see here following microglial manipulation is an acceleration of memory development leading to the precocious emergence of memory, rather the opposite. It is important to note that infants do have the ability to form memories and retain memory for some time after which has been shown by many groups and as we have shown with recall tests 1d and 3d post-training but have forgotten by the onset of infantile amnesia (8d post training) at P25 so what we are seeing is not the precocious emergence of memory development.

Furthermore, work by (Ramsaran et al. 2023) does not investigate infantile amnesia per se/ Rather, they demonstrate imprecise memory formation in juvenile mice illustrated by high levels of memory generalisation between contexts, and that more precise “adult-like” memories can be formed through manipulation of neuronal excitability and increased PNNs. However, they do not test longer retention intervals more than 1d so its not clear whether these memories last, or if there is any change in infantile amnesia.

Importantly, what they also demonstrate is a developmental increase in PNNs between P20 and P24. If we assume that we prevent that normal trajectory of development and maturation then one interpretation is that we prevent this increase of PNNs at P24 which is an important step in memory maturation. This idea is also supported by new data we have added the level of microglial activation which is low at P20 and P22, when mice still remember but is increased at P25 when amnesia has occurred (Figure 1). By inhibiting microglia we may prevent this increase in activity and infantile amnesia. We have now discussed this in the discussion.

        * Moreover, the authors report that minocycline leads to an increase in engram size in the DG and AMG and conclude that the observation that engrams are similarly affected in different regions points to a common mechanism behind infantile forgetting.  Again, this seems somewhat contradictory to past work (Ramsaran et al. (2023)), where the development of sparse engrams is thought to drive memory development.  The authors should try to reconcile these divergent results.

The larger engram size we see is consistent with our previous publication (Power et al ’23) where we saw larger engram size in male MIA offspring, which also did not display infantile amnesia. Similar to our above argument, (Ramsaran et al. 2023) do not demonstrate any long term retention of the memory, it is not clear whether in their case the more sparse engram, while it does lead to adult-like precision, also leads to better memory retention. Similar microglial driven mechanisms may also still lead to amnesia in those mice despite the enhanced precision.

While we do not test changes in generalisation extensively as it is beyond the scope of the current manuscript, we do not see differences between control and minocycline-treated mice tested in context B (Fig S1). For these reasons it is difficult to directly compare this result. Additionally, we only see a larger engram in the BLA and (Ramsaran et al. 2023) are manipulating engram size in the CA1.

        * The final results section attempts to relate the experimental findings in healthy developing mice to understand cognitive deficits in MIA mice.  Here, the authors show that minocycline reinstates IA in MIA mice (that "naturally" do not show IA).  However, this result does not seem logically consistent with the previous data.  Namely, if MIA leads to reduced microglial activity in developing mice which prevents IA, then why would administering a drug that reduces microglial activity further lead to the emergence of IA in these mice?  I believe the authors try to address this point in their discussion, but the logic of using minocycline to understand the neurobiological mechanisms that prevent IA in MIA needs to be explicitly explained in the results section to improve coherence and readability

We have added to the results section our logic for using minocycline and the selected developmental time window which we hope improves coherence and readability.

References :

Dalmau, I. *et al.* (1998) ‘Development of microglia in the postnatal rat hippocampus’, *Hippocampus*, 8(5), pp. 458–474. Available at: https://doi.org/10.1002/(SICI)1098-1063(1998)8:5<458::AID-HIPO6>3.0.CO;2-N.

Orłowski, D., Sołtys, Z. and Janeczko, K. (2003) ‘Morphological development of microglia in the postnatal rat brain: A quantitative study’, *International Journal of Developmental Neuroscience*, 21(8), pp. 445–450. Available at: https://doi.org/10.1016/j.ijdevneu.2003.09.001.

Jin B, Gongwer MW, Kearney BP, Ohanian L, Holden-Wingate L, Le B, Darmawan A, Nakayama Y, Mora SAR, DeNardo LA. A developmental brain-wide screen identifies retrosplenial cortex as a key player in the emergence of persistent memory. bioRxiv [Preprint]. 2024 Nov 8:2024.01.07.574554. doi: 10.1101/2024.01.07.574554. PMID: 38260633;
